# 1/f^2^ Characteristics and Isotropy in the Fourier Power Spectra of Visual Art, Cartoons, Comics, Mangas, and Different Categories of Photographs

**DOI:** 10.1371/journal.pone.0012268

**Published:** 2010-08-19

**Authors:** Michael Koch, Joachim Denzler, Christoph Redies

**Affiliations:** 1 Department of Computer Science, Friedrich Schiller University, Jena, Germany; 2 Institute of Anatomy I, School of Medicine, Friedrich Schiller University, Jena, Germany; University of Regensburg, Germany

## Abstract

Art images and natural scenes have in common that their radially averaged (1D) Fourier spectral power falls according to a power-law with increasing spatial frequency (1/f^2^ characteristics), which implies that the power spectra have scale-invariant properties. In the present study, we show that other categories of man-made images, cartoons and graphic novels (comics and mangas), have similar properties. Further on, we extend our investigations to 2D power spectra. In order to determine whether the Fourier power spectra of man-made images differed from those of other categories of images (photographs of natural scenes, objects, faces and plants and scientific illustrations), we analyzed their 2D power spectra by principal component analysis. Results indicated that the first fifteen principal components allowed a partial separation of the different image categories. The differences between the image categories were studied in more detail by analyzing whether the mean power and the slope of the power gradients from low to high spatial frequencies varied across orientations in the power spectra. Mean power was generally higher in cardinal orientations both in real-world photographs and artworks, with no systematic difference between the two types of images. However, the slope of the power gradients showed a lower degree of mean variability across spectral orientations (i.e., more isotropy) in art images, cartoons and graphic novels than in photographs of comparable subject matters. Taken together, these results indicate that art images, cartoons and graphic novels possess relatively uniform 1/f^2^ characteristics across all orientations. In conclusion, the man-made stimuli studied, which were presumably produced to evoke pleasant and/or enjoyable visual perception in human observers, form a subset of all images and share statistical properties in their Fourier power spectra. Whether these properties are necessary or sufficient to induce aesthetic perception remains to be investigated.

## Introduction

The basis of aesthetic judgment remains elusive. Properties that can be expressed in scientific or mathematical terms and are common and unique to most (or possibly all) aesthetic images, including visual art, have yet to be identified. Indeed, it is still controversial whether such universal criteria for aesthetic judgment exist at all. On the one hand, it has been argued that the appreciation of aesthetic artworks relies on cultural variables that differ substantially between styles of art and cultural provenance (for example, see [1], [2]). On the other hand, some artists, philosophers, and, more recently, neuroscientists have postulated that aesthetic judgment is based on principles, which are shared by human beings, independent of their cultural background [3]–[9].

As a first step in the search for properties that distinguish aesthetic images from other image categories, we and other researchers have studied statistical properties of subsets of aesthetic images and found that they possess, on average, scale-invariant statistical properties in the Fourier domain [10]–[13]. Specifically, the radially averaged spectral amplitudes in art images fall according to a power-law with increasing spatial frequency, similar to 1/f noise (or according to 1/f^2^, if spectral power is plotted instead of amplitude, as done in the present study; f  =  spatial frequency). 1/f^2^ characteristics have been found previously in images of natural scenes [14]–[17]. These findings imply that both art images and natural scenes possess fractal-like properties, i.e., the amplitudes of the spatial frequencies remain constant if one zooms in and out of the images (for reviews, see [18], [19]). Because the processing of information in the human visual system is efficiently adapted to viewing natural scenes [20]–[23], it has been argued that artists use 1/f^2^ characteristics in their artworks because these properties confer inherent aesthetic value [24] or are a corollary of aesthetic features in artworks [9].

1/f^2^ characteristics apply, on average, to a wide range of artworks, including art of different techniques and styles, both abstract and figurative, from the Western hemisphere [10]–[12] and of Eastern provenance [25]. Fractal-like properties have been demonstrated also for special types of abstract art, notably for the drip paintings of Jackson Pollock [26]–[28]. In the present study, we asked whether other categories of man-made images that are presumably created to evoke pleasant or enjoyable visual perception in humans also display 1/f^2^ characteristics in the Fourier domain. To answer this question, we extended our previous analysis [11], [12] to political cartoons and graphic novels (Japanese mangas and comics of Western provenance).

While computing the radially averaged (1D) power spectrum was in line with previous research and results by others [10], [14]–[17], 1/f^2^ characteristics are neither necessary nor sufficient to induce aesthetic perception [11]. Notably, images with 1/f^2^ characteristics that are not necessarily aesthetic can be produced artificially [29], [30]. Hence, such a property cannot be interpreted as an exclusive feature of aesthetic images. Because radially averaging the power spectrum of an image means loss of information, the next natural step taken in our investigation is to study the complete (2D) Fourier spectrum and to search for properties that are common to visually pleasing images in such a representation. In order to define the relation between 2D Fourier spectral properties and different categories of man-made images more closely, we compared man-made images with photographs of real-world objects (natural scenes, plants, simple objects and faces).

Currently, we are looking for common second-order properties of different categories of images in general and restrict our investigation to the power spectrum. The phase information of an image was excluded from the analysis. Higher-order statistics should be studied in future investigations of the different image categories.

## Results

### Experiment 1: Analysis of radially averaged (1D) Fourier power spectra of cartoons, comics and mangas

**Figure 1:**
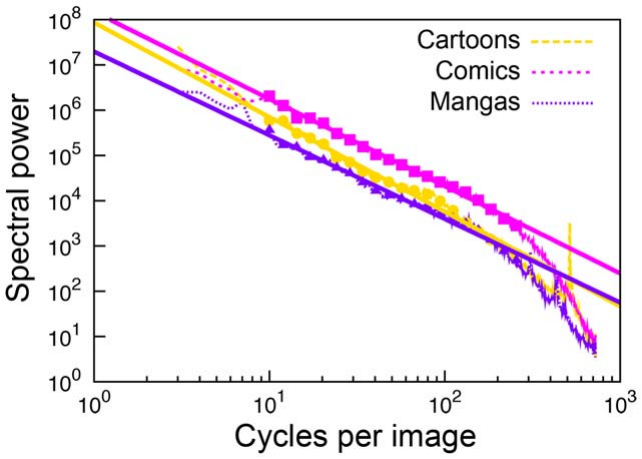
Examples of log-log plots of radially averaged 1D power spectra. One example each is given for the image categories of cartoons, comics and mangas (thin colored lines). Note that log power falls nearly linearly with increasing log frequency. The squares represent the binned points (10–256 cycles/image) that were used for fitting the straight lines (thick colored lines) for each image. The slope of the lines was −2.09, −1.93, and −1.85 for the cartoon, comic and manga, respectively.

Previous studies have shown that the radially averaged spectral amplitudes of monochrome art images fall roughly according to a power-law with increasing spatial frequency (1/f^2^ characteristics, see Introduction). In the present study, we extended this type of analysis to political cartoons, Japanese mangas and comics of Western provenance. Examples for the log-log plots of radially averaged power spectra are shown in Figure 1. Mean slope values for the three image categories are given in Table 1 and demonstrate that, like visual art and natural scenes, all three image categories possess 1/f^2^ characteristics in their power spectra. The mean slope values of the three image categories are significantly different from those of scientific illustrations (p<0.001), face photographs (p<0.001) and household objects (p<0.001) (Table S1). These results demonstrate that other man-made images that are produced to evoke pleasant perception in humans, also possess 1/f^2^ characteristics.

**Table 1:** Slope of fitted line in log-log plot of radially averaged spectral power and deviation of the data from the fitted line.

Image category	n	Slope	Deviation
Monochrome art^1^	200	−2.07±0.37	0.015±0.018
Monochrome portraits^2^	306	−2.12±0.30	0.018±0.019
Natural scene photographs^1^	208	−2.03±0.33	0.010±0.010
Face photographs^2^	3313	−3.54±0.15	0.016±0.021
Scientific illustrations^1^	209	−1.57±0.32	0.019±0.021
Object photographs^1^	179	−2.75±0.28	0.010±0.010
Plant photographs^1^	206	−2.90±0.38	0.010±0.013
Cartoons^3^	230	−1.99±0.24	0.017±0.016
Comics^3^	247	−2.04±0.25	0.021±0.024
Mangas^3^	244	−2.08±0.18	0.010±0.008

Values represent mean ± SD (n, number of images analyzed for each category). For pairwise significance testing, see Table S1.

1 Data from Redies et al. (2007a).

2 Data from Redies et al. (2007b).

3 Slopes were calculated without lanczos/sinc function (see Methods), according to previously used methods of calculation (Redies et al., 2007a,b).

### Experiment 2: Principal component analysis of the 2D Fourier power spectra

As a next step in our analysis, we asked whether differences exist between the 2D power spectra of the man-made and real-world image categories.

The basis for our analysis is a generative model of the 2D power spectrum. We assume that the 2D power spectrum ***F*** (represented as a vector ***f***) of an individual image is generated by a linear model ***f***  =  ***W*** ^T^ ***s.*** Thus, the 2D power spectrum of an image is represented by a point ***s*** in the space spanned by a given set of basis spectra ***W***. Examples of the calculated 2D power spectra for each image category are given in Figures S1 and S2.

**Figure 2:**
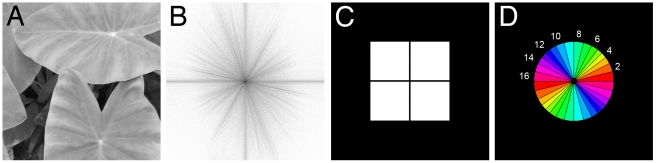
Analysis of the power spectrum. (A) Example of a photograph of a plant and its corresponding power spectrum (B). (C) Mask used for PCA analysis. To remove artifacts in the power spectrum, black parts containing high frequencies (larger than 128–181 cycles/image) and horizontal/vertical orientations were ignored in the PCA analysis. (D) Illustration of the sectors used to calculate gross anisotropy in the images. Corresponding sectors of the symmetric power spectrum are displayed in the same color. Sector numbers correspond to those shown in Figure 5.

Principal component analysis is used to reconstruct the “basis power spectra”, as done before for the space of images [31] or the so-called Eigenfaces as a representation of the space of face images [32], [33]; it is widely used in visual studies on the representation of shape and structural appearance in images. For example, such an approach has been successfully used to categorize photographs of urban and natural scenes [34]. Given such a linear model of the generative process for 2D power spectra, we can investigate whether images of different categories are located in different subspaces of the spanned space of the 2D power spectrum. For technical reasons, we restrict our investigation to the first 15 basis vectors given by the 15 largest eigenvalues of the PCA analysis. Because high frequencies (>126–181 cycles/image) as well as horizontal and vertical orientations likely contained artifacts, we neglected them in the initial PCA (see Methods and Fig. 2C).

Although the number of images available in our data set for PCA is not sufficient for a full rank covariance matrix computation, we were able to demonstrate that the power spectrum exhibits differences between image categories, including man-made images. With the procedure, we are in line with current investigations in this area, like the work by Torralba et al. [34], who did a PCA of the power spectrum using 5000 images of size 256×256.

**Figure 3:**
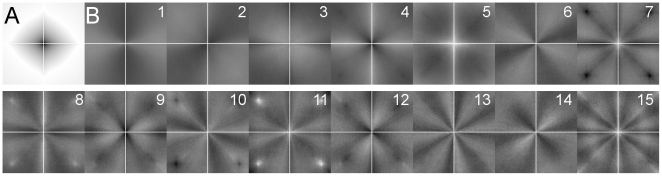
Principal components of the power spectra of all image categories. The mean power spectrum for all categories (A) and the first 15 principal components are shown with image normalization for each image (B). To avoid artefacts, the power spectra were analyzed only in the parts that were left uncovered by the mask displayed in Fig. 2C.

Figure 3 displays the mean power spectrum (Fig. 3A) and the first 15 principal components (PCs; Fig. 3B) for all 1500 spectra analyzed (150 randomly selected images per image category). The mean power spectrum displays a smooth gradient from high frequencies to low frequencies that is rather uniform across angular directions. The PCs show a higher degree of anisotropy of gradients across orientations. A similar set of PCs has been obtained previously for a large set of natural and urban scenes [34].

Because the number of images available for principal component analysis was not sufficient for a full rank covariance matrix computation, we also calculated the first 15 PCs for 2D power spectra that were reduced in their resolution from 256×256 pixels to 16×16 pixels, to obtain a more complete covariance matrix. The first eight PCs had a similar appearance in both calculations, but the ninth to fifteenth PCs displayed an increasingly noisy structure in the 16×16 pixel calculation (data not shown).

In order to quantify how close two image categories are in the 15D eigenspace, the covariance matrix was extracted and the mean Mahalanobis distance [35] was calculated for each pair of image categories (Table S2). Thereby, the covariance matrix and the mean of all coefficients of one image category define the specific distance metric of this category. Hence, the columns of Table S2 represent the distance of the mean coefficients of every category to one specific distance metric of a single category.

**Figure 4:**
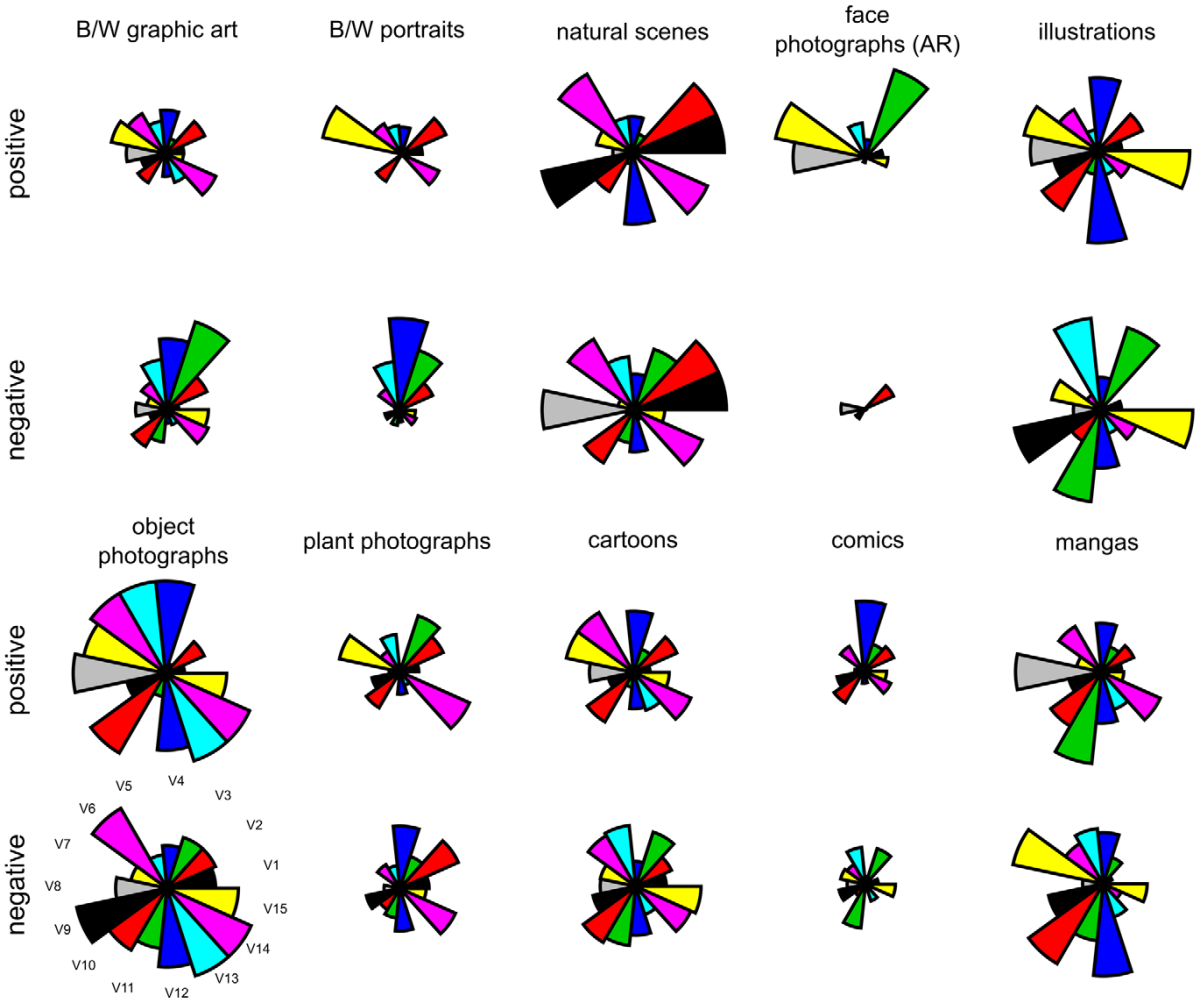
The mean positive and negative coefficients of the first 15 principal components. Results for each image category are displayed in a radar plot. Sector length represents the coefficient strength normalized across all coefficients. The numbers of the sectors at the bottom left of the figure indicate the order number of the principal components. Radar plots are similar for B/W graphic art and B/W portraits, but show relatively large differences for all other image categories.

The Mahalanobis distance does not take into account the angle between the coefficient vectors for each image in the 15D space. To obtain a better idea of the differences in the coefficients between image categories, Figure 4 visualizes the mean coefficients of the first 15 PCs of all categories as radar plots for the analysis at a high resolution (256×256 pixels) of the Fourier spectra. We chose to plot positive and negative coefficients separately to obtain a better impression of the variance of the coefficients. The resulting plots show large differences in the first 15 PCs between all image categories, except for graphic art and portraits, which are more similar to each other than to most of the other image categories.

The mean number of coefficients that differ significantly (p<0.001) between all pairs of image categories was smaller for the original images (4.0±2.5 S.D.) than for their respective power spectra (6.1±2.2 S.D.), suggesting that the Fourier spectra allow for a better separation of the image categories than the original images.

Taken together, the results from the PCA demonstrate that the monochrome art images (graphic art and portraits) occupy subspaces in the 15D eigenspace. This implies that they exhibit specific features in their Fourier spectra that allow separating them, at least in part, from the other image categories. In future studies, a greater understanding of the higher-order geometry of each image category may eventually lead to a more complete separation in feature space.

### Experiment 3: Analysis of isotropy in the 2D Fourier power spectra

**Figure 5:**
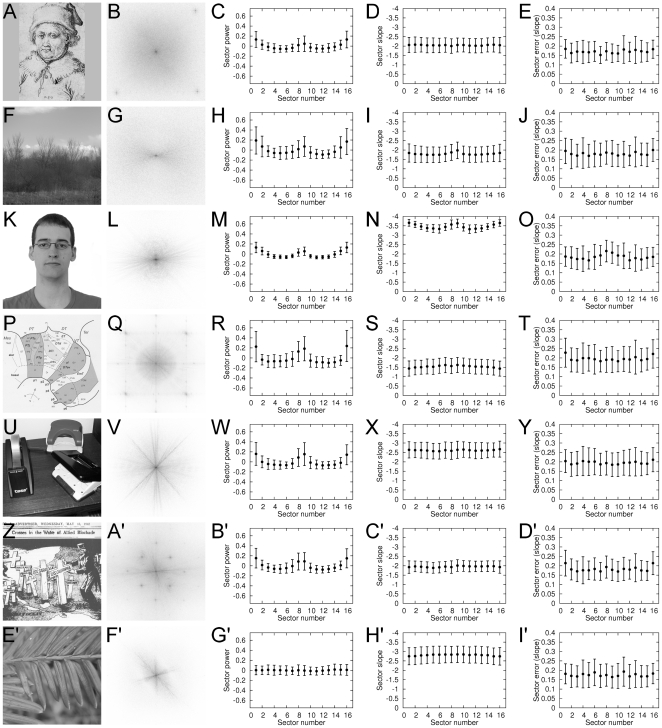
Frequency signature analysis of samples from different image categories. The first column displays a sample image of monochrome graphic art (A), natural scenes from the Groningen database (F), face photographs (K), scientific illustrations (P), object photographs (U), cartoons (Z) and plant photographs (E'). The second column shows the corresponding power spectra (B, G, L, Q, V, A', F'). The low spatial frequencies are represented at the center and darker shades represent higher power. The sector parts shown schematically in Fig. 2D were analyzed. For all images in the same category, the three columns on the right side represent the mean power for each sector of the power spectrum (C, H, M, R, W, B', G'; for numbering of the sectors, refer to Fig. 2D); the mean sector slope values (D, I, N, S, X, C', H'); and the mean sector error of the line approximation (E, J, O, T, Y, D', I'). The whisker plots represent mean values and their standard deviation. The image shown in A is an engraving by the 15th century artist Martin Schongauer and was reproduced with permission from “Das Berliner Kupferstichkabinett”, Akademischer Verlag, Berlin, 1994 (inventory number: 916-2; © Staatliche Museen zu Berlin, Kupferstichkabinett). F displays an example from the Groningen database of natural scenes [38]. K is a photograph similar to those of the AR face database [45] and shows one of the authors (M.K.), to avoid conflicts with the rights of the persons photographed for the AR database. The scientific illustration in P, the object photograph in U, and the plant photograph in E' are from the study by Redies et al. [12]. Images are reproduced with permission from the authors. Z displays a propaganda cartoon from the Japan Times (from the year 1942; downloaded from Wikimedia Commons).

In view of the obtained differences between the image categories, we next attempted to define specific properties of the Fourier spectra for each image category. In preliminary experiments, we observed that one key difference between the power spectra of the different image categories is their general shape, which can be classified as isotropic vs. anisotropic in the Fourier domain (Figs. 2B; 5B,G,L,Q,V,A'; S1, S2). In the present work, we determined anisotropy both of mean power and of power gradients.

For mean power, anisotropic implies that power averaged across frequencies varies with the orientation in the power spectrum of an image (“power anisotropy”). This value was determined because natural scenes generally contain higher proportion of power at cardinal orientations (horizontal and vertical) than at oblique orientations, although orientational power can vary considerably between individual images [36]. We asked whether artists mimic or change this bias in their artworks. The antonym, isotropic, implies that spectral power is uniform across all orientations in the power spectrum.

For power gradients, anisotropic means that the gradients from low to high frequencies vary in their steepness (or slope) with the orientation in the power spectrum of an image (“slope anisotropy”). This value was determined in order to assess whether the observed scale-invariant power spectra of natural scenes and aesthetic images varied with regard to different orientations.

Our next step was to look for a representation of the power spectrum that makes classification into isotropic/anisotropic possible. In contrast to some previous work by others, for example the frequency signature approach of Torralba and Oliva [34], we did not fit a specific model to the averaged power spectra of a class of images in our work, but we computed mean power and the slope of a straight line fitted to the log-log power spectrum, across different orientations for individual images, as previously done for natural scenes by van der Schaaf and van Hateren [36]. To do this, we divided the power spectrum into 32 equal sectors (Fig. 2D). However, because upper and lower parts of the Fourier spectra are identical, the analysis was restricted to the upper 16 sectors. The average mean power and the average slope value for each sector were calculated. Directional slope values were calculated in the same way as the rotational averages (see Methods; compare to Fig. 1). To assess how well a line can approximate the shape of the log-log power spectrum for the individual sectors, we computed the error of the fit for individual sectors.

We then plotted the resulting power and the slope values over the sectors and call this representation the frequency signatures of an individual image. For an ideal isotropic spectrum, the frequency signature will be a constant function. For an anisotropic one, it will be a function that depends on the change of the power or the slope over different orientations in the power spectrum.

The frequency signatures of individual images from one image category were used as the basis to compute the average frequency signatures of this category. In Figure 5, different sample images (first column in Fig. 5) and the average signatures for power (third columns) and slope values (forth columns) are given for each image category. The reader can observe that the frequency signatures correspond to the visual appearance of the 2D power spectra (compare to examples in Figs. S1, S2) with respect to isotropy vs. anisotropy of power and slope values.

Results reveal that photographs of natural scenes (Fig. 5F–J), faces (Fig. 5K–O) and objects (Fig. 5U–Y) contain a higher proportion of power in cardinal orientations (horizontal: sectors 1 and 16; vertical: sectors 8 and 9; compare to numbering in Fig. 2D) than in oblique orientations. Similar observations were made for real-world photographs of natural and urban scenes [34], [36], [37]. A bias of mean power for cardinal orientations was also observed for the aesthetic image categories (monochrome graphic art [Fig. 5A–E] and cartoons [Fig. 5Z–D']) and scientific illustrations (Fig. 5P–T). Plants that were photographed from above are the only image category that shows similar mean power values in all directions of the power spectrum (Fig. 5E'–I'), as expected from the lack of a viewpoint bias in these images.

**Figure 6:**
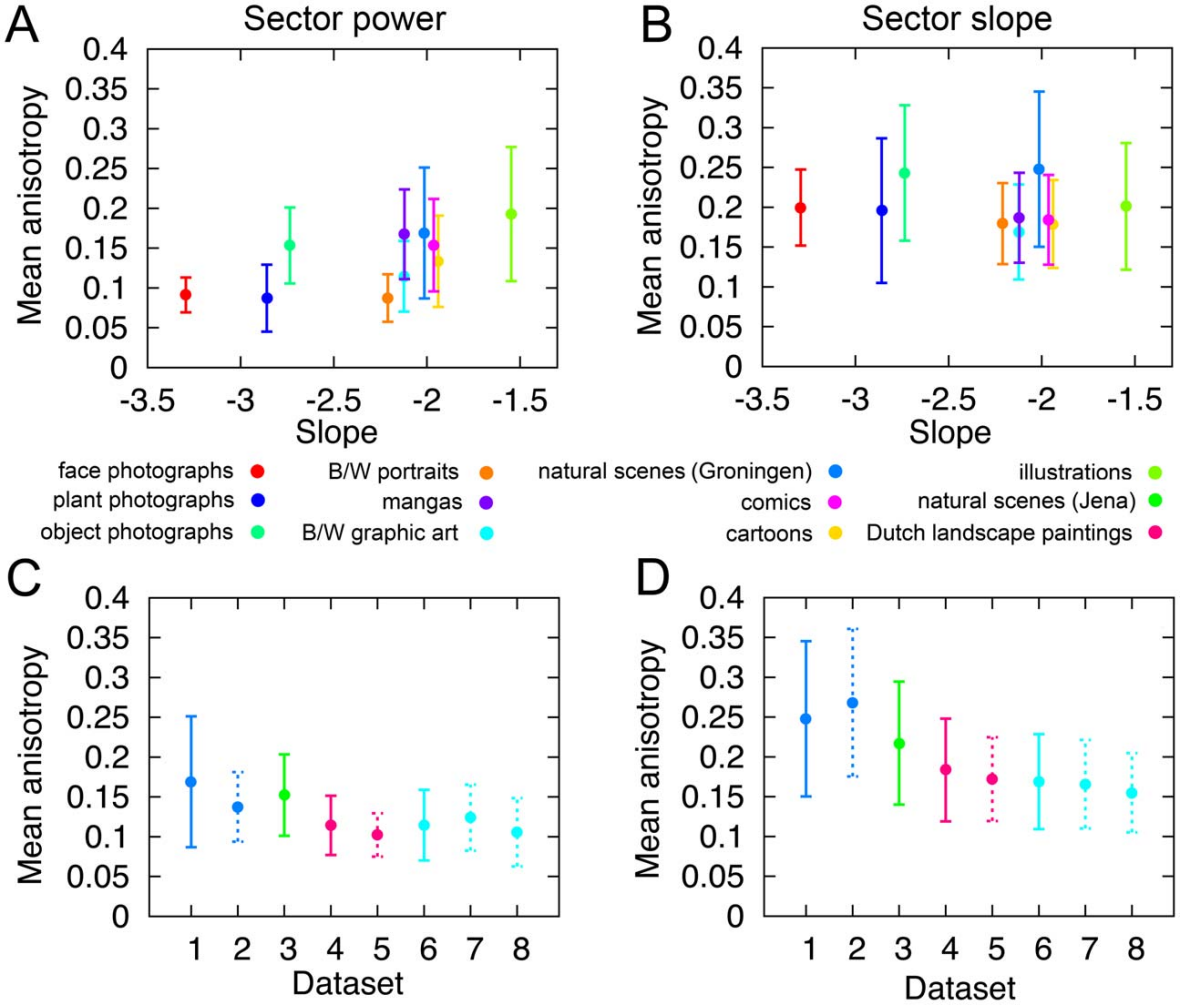
Quantitative results of measuring power and slope anisotropy in the power spectra of the different image categories. For each category, mean power anisotropy values (A, C) and mean slope anisotropy values (B, D) were calculated on the basis of the standard deviations of the mean power values and of the slope values, respectively, for the 16 radial sectors in each image (compare to Figs. 2D, 5). In A and B, results are plotted as a function of the radially averaged slope values for each image category (see Tab. 1). In C and D, data for the following datasets are plotted: Dataset 1, the Groningen database of natural scenes; dataset 2, the 30 images of the Groningen database, matched in image content to 30 images of the Dutch landscape drawing and print database (dataset 5); dataset 3, large-vista natural scene database (Jena); dataset 4, 104 Dutch landscape drawings and prints; dataset 5, the 30 images of the Dutch landscape drawing and print database, matched in image content to the 30 images of the Groningen database (dataset 2); dataset 6, monochrome graphic art; dataset 7, 50 images from the monochrome graphic art database with strong perspective or depth cues; and dataset 8, 50 images from the monochrome graphic art database with weak or no perspective or depth cues. The whisker plots represent mean values and their standard deviation.

The mean standard deviation over all image sectors of an image was taken as a measure of anisotropy. Figure 6 displays mean anisotropy values for the different image categories. For power anisotropy (Fig. 6A), there is no systematic difference between the categories of real-world photographs (faces, plants, objects, natural scenes) and the man-made image categories (monochrome art and portraits, cartoons, graphic novels and illustrations). For the different subcategories of art images, mean power isotropy values are similar for different centuries, countries of origin and techniques (Table S3, left column). However, power anisotropy is significantly higher for artworks depicting buildings than for other subject matters.

We next studied anisotropy of the slopes of the power gradients. For natural scenes and photographs of faces, slope values are significantly higher for horizontal orientations and vertical orientations than for oblique orientations (p<0.001; Fig. 5I,N). Differences between mean slopes in cardinal and oblique orientations are less prominent or absent in the other image categories, including monochrome graphic art (Fig. 5D), monochrome portraits, cartoons (Fig. 5C'), comics and mangas (data not shown).

The mean anisotropy of the slope values is plotted in Figure 6B for each image category (for results of pairwise significance testing, see Table S1). Slope anisotropy was higher for photographs of natural scenes, objects and faces, as well as scientific illustrations than for art images, cartoons, comics, and mangas (p<0.05). Photographs of faces showed slightly higher slope anisotropy values (0.20±0.05 S.D.) than portraits by artists (0.18±0.05 S.D., p<0.001). For the monochrome art dataset analyzed in this study, slope anisotropy values are generally similar across different centuries, country of origin, techniques and subject matters, with few exceptions (etchings, drawings and living matters; Table S3).

The differences in anisotropy between the image categories may be due to viewpoint bias or differences in stationarity. For example, photographs of natural scenes are often captured at eye level and display large areas of horizon. These features induce stronger power anisotropies [34], [36], [37] but can potentially introduce also stronger slope anisotropy. The photographs of the Dutch countryside taken by van Hateren and van der Schaaf [38] lack large vistas but include bodies of water or plants at close range, which may also cause the anisotropic spectra. In contrast, artists are free to choose any angle of perspective and, consequently, their works may be more isotropic. Despite this freedom, the power anisotropy of aesthetic images resembles that of natural scenes (Fig. 6A). In contrast, slope anisotropy in visually pleasing images (artwork, cartoons, and graphic novels) is generally lower than in the other image categories (Fig. 6B). To assess the potential influence of viewpoint choice on the measured slope isotropy values, we carried out three additional types of analyses.

First, we determined slope anisotropy for a novel set of 198 large-vista photographs of Rocky Mountain and Norwegian landscapes (dataset 3 in Fig. 6C, D) and confirmed that mean slope anisotropy in these photographs (0.22±0.08 S.D.) was significantly larger than in artworks (0.17±0.07 S.D., p<0.001), although it was smaller than for the Groningen database (0.25±0.10 S.D.; p<0.001; dataset 1 in Fig. 6D).

Second, for the dataset of graphic art, we compared 50 images of artworks that contained strong perspective lines and depth cues (dataset 7 in Fig. 6C, D), to 50 images of artworks that contained no or only weak perspective and depth cues (dataset 8 in Fig. 6C, D). Results revealed that slope anisotropy values did not differ significantly between the two types of artworks (Fig. 6D).

Third, we compared the Groningen database of natural scenes (dataset 1 in Fig. 6C, D) to 104 monochrome drawings and prints of Dutch landscapes (dataset 4 in Fig. 6C, D). Slope anisotropy values for the landscape images painted by artists were significantly lower (0.18±0.06 S.D.) than values for the Groningen natural scenes database (0.25±0.10 S.D.; p<0.0001) and resembled those of the other art categories. Because the type of scenery contained in the two datasets did not match completely, 30 images with similar content were selected from the two databases. The 30 matched images from the database of Dutch landscape paintings (mean anisotropy 0.17±0.05 S.D.; dataset 5 in Fig. 6D) had lower slope anisotropy values than the 30 matched images from the Groningen database (0.27±0.09 S.D.; p<0.0001; dataset 2 in Fig. 6D), in accordance with the overall difference between the two image databases. Differences in power anisotropy showed similar overall tendencies (Fig. 6C).

Taken together, these results indicate that the lower degree of slope anisotropy found in artworks cannot be explained on the basis of viewpoint bias or less use of perspective cues alone. Rather, they suggest that artists use Fourier spectra in their artworks that tend to be more isotropic with respect to the ratio between high and low frequency power than those of corresponding real-world images. In contrast to this result from the slope anisotropy measurements, no systematic differences between the two types of images were found in the power anisotropy measurements. The significance of this discrepancy remains to be explored further.

## Discussion

### Scale invariance in the power spectra of man-made images

In an attempt to define statistical properties of aesthetic visual art, we and others have described previously that, on average, art images of different techniques, style, subject matter and cultural background (Western and Eastern provenance) share scale-invariant (fractal-like) statistics in their radially averaged Fourier spectra [10]–[13], [25]. Scale invariance is suggested if slope values of log-log plots of radially averaged Fourier power versus spatial frequency are around −2 (1/f^2^ characteristics). In this respect, art images resemble natural scenes, which also possess 1/f^2^ characteristics (see Introduction). Similar findings were published for images of architecture (for a review, see [39]), American sign language [40] and music [41], [42].

In the present study, we extend these observations to other categories of monochrome man-made images, cartoons and graphic novels (Japanese mangas and comics of Western origin). Like art, these images are created for viewing by humans and most of them are designed to evoke pleasing or enjoyable perception upon viewing. It is therefore noteworthy that the slopes of cartoons and graphic novels are around −2 as well (Table 1). Scientific illustrations, which are primarily produced to convey content and are not necessarily aesthetic or pleasing, have slope values significantly higher than −2 [12]. Photographs of faces, simple objects and plants have slope values significantly lower than −2 (Table 1) [12], [34]. Fernandez and Wilkins (2008) showed that the spectral components of comfortable images resemble those of natural scenes while uncomfortable images have energies that are disproportionately higher within two octaves of 3 cycles per degree [43]. Taken together, these results suggest that a wide variety of visually pleasing images share specific statistics in their radially averaged power spectra [13].

### Discrimination of image categories by principal component analysis

As a next step in our analysis, we asked whether the full 2D power spectra of each image category have a characteristic structure that allows distinguishing them from other image categories. After extracting the first 15 principal components (PCs) of the power spectra by principal component analysis (PCA [44]) from all 1500 images analyzed in the present study, we found that each image category occupies a disjunct subspace (eigenspace) in the space spanned by the 15 PCs, with the exception of monochrome art and art portraits, which had largely overlapping eigenspaces. It should be noted that PCA is a linear transformation and we neglected the phase part in our analysis. Future work with non-linear transformations that explicitly take account of structural (phase) information may elucidate further properties that characterize aesthetic and other visually pleasing images and possibly allow a better distinction between this type of man-made images and real-world photographs. Our study should thus be viewed as a first step in this type of analysis.

### Anisotropy of the 2D power spectra

Because the PCA indicated that the different image categories each have characteristic power spectra, we next asked what these differences might be. Preliminary inspections of the power spectra indicated that those of art images may be more isotropic. We therefore measured two types of anisotropy directly in the individual power spectra by a sector analysis (Figs. 5, 6). First, we determined the anisotropy of mean directional power. Results reveal that in both visually pleasing images (artworks, cartoons, comics, and mangas) and real-world photographs (natural scenes, faces and objects) power is concentrated in cardinal orientations. Thus, artists do not take advantage of their freedom to deviate from having higher power in cardinal orientations.

In contrast, slope anisotropy is generally lower in art images, cartoons and graphic novels than in most of the other real-world and man-made categories (face photographs, natural scenes, scientific illustrations, and photographs of objects; Fig. 6B). Low slope anisotropy in art images is independent of cultural variables, such as century, techniques, country of origin and subject matter (Tab. S3). However, not all images of natural objects display a high degree of slope anisotropy. For example, the power spectra of plant photographs are relatively isotropic, both for mean power and power slopes. Because these images are not necessarily aesthetic, low slope anisotropy alone does not seem to be sufficient to induce aesthetic perception.

One possible reason for a higher degree of slope anisotropy in natural scenes is a bias for horizontal and vertical orientations due to the viewpoint taken by the photographer. However, viewpoint bias alone cannot explain the higher slope isotropy in art images, for the following reasons: (1) artistic portraits display higher slope isotropy than face photographs; (2) landscape paintings are, on average, more isotropic than landscape photographs, and (3) artworks, which are composed with strong perspective and depth cues, are as isotropic as artworks with weak or no perspective cues (Fig. 6D).

We conclude that, in addition to 1/f^2^ characteristics, artists tend to shift the power spectra of real-world images to more isotropic ones in their artworks. Other types of visually pleasing man-made images, such as cartoons and graphic novels, also show a tendency towards higher slope isotropy.

It remains to be investigated in a more systematic way whether isotropic 1/f^2^ characteristics represent a universal property of all aesthetic or visually pleasing images and whether they are necessary or sufficient to elicit an aesthetic response in human observers. Also, the statistical properties measured by us should be directly related to aesthetic ratings by human observers within a category of images (e.g., photographs), in order to scrutinize their relevance for aesthetic judgment. Moreover, there is significant overlap of the image types in their common eigenspace, and the models of directional isotropy do not achieve a complete separation between the categories of aesthetic and non-aesthetic images. Despite these caveats, our results provide evidence that artworks constitute a subset of all images with specific properties that tend to deviate from real-world objects and scenes. Moreover, artists seem to produce aesthetically pleasing images by making preferential use of specific statistical properties that can be extracted by lower-level perceptual mechanisms, similar to object categorization in natural scenes by gist perception [34].

## Materials and Methods

### Image data

For analysis, we used sets of previously analyzed image databases [11], [12] and novel image databases of cartoons, graphic novels (comics and mangas), large-vista natural scenes, and Dutch landscape drawing and prints. The previously analyzed data include the following databases:

#### Photographs of faces

3313 images from the AR face database [45]. The AR face database contains color images of 126 people with different facial expressions, illumination conditions and occlusions, photographed on a uniformly bright background. Image size was 768×576 pixels. Images were converted to grayscale values. Centered passport-type details of 576×576 pixels (AR face database) were cut from each image for analysis.

#### Natural scene database

A dataset of 208 images selected from the Groningen natural scene database [38]. The selected photographs did not contain buildings and other man-made structures. As described previously [12], centered details of 1024×1024 pixels were cut from the original monochrome images of size 1536×1024 pixels.

#### Monochrome graphic art databases

Datasets of 200 monochrome graphic art images [12] and 306 monochrome portraits by artists [11]. The artworks were of Western provenance and represented a large variety of graphic styles, subject matters, techniques, centuries and artists. The vast majority was created by well-known artists and collected by prestigious museums. Images were scanned from high quality art books by a calibrated scanner in 8-bit grayscale. Largest possible square details from the art images or the complete images padded according to square ones by adding a uniform gray border were reduced to a resolution of 1024×1024 pixels by appropriate software (Photoshop, Adobe, Mountainview, CA).

#### Photographs of landscapes, objects and plants

179 photographs of household or laboratory objects and 206 photographs of plants or parts of plants. The generation of these datasets was described previously [12]. The photographs were obtained with a 4-megapixel digital camera (Digital Ixus 400, Canon, Tokyo, Japan). Square details of maximal possible size were taken from the digital images and converted to grayscale images (1024×1024 pixels). By the same method, a database of 198 large-vista photographs of Rocky Mountain and Norwegian landscapes was generated for the present study.

#### Scientific illustrations

209 scientific illustrations (anatomical illustrations, schematic diagrams and recordings) from different textbooks on genetics, anatomy or neuroscience [12]. The images were scanned and processed in the same manner as the monochrome graphic art images. The illustrations did not contain photomicrographs of natural objects or scenes.

For the present study, the following sets of images were digitized from books on a scanner that was calibrated as described previously [11]. Monochrome images were scanned in 8-bit gray scale. Care was taken to include a large variety of styles and artists in each image category. For all images, square details of maximal possible size were obtained and reduced to 1024×1024 pixels.

#### Cartoons and graphic novels (mangas and comics)

230 monochrome (grayscale) political cartoons from the 20th century were scanned from books that contained collections of cartoons from different countries and cartoonists. Typically, each cartoon depicted of a single scene. 244 monochrome Japanese mangas were scanned from 20 different contemporary manga books purchased in Japan in the year 2007 and one review book on mangas. 247 monochrome 20th century graphic novels of Western provenance were scanned either from various books on work by single artists or from collections of comics from different countries and artists. For graphic novels, entire pages were digitized and cropped to square details of maximal possible size. Typically, for mangas and graphic novels, each image consisted of several panels depicting different scenes.

#### Dutch landscape drawing and prints

104 monochrome (grayscale) images of Dutch landscape drawings and prints were scanned from five artbooks on 15^th^ to 19^th^ century Dutch landscape paintings. The database contained large-vista views of landscapes with wide horizons, skies of bodies of water as well as close-up views of trees and vegetation without large horizons.

### Fourier transformation

Image analysis was performed using Matlab and C++. For rotational averaging and anisotropy/model analysis, the images were resized by bicubic interpolation to a resolution of 1024×1024 pixels (Matlab routine). For all PCA investigations (C++), images were resized to 512×512 pixels, due to memory and computation time constraints. The power spectrum (amplitude squared) of each input image was obtained by computing the 2D Fast Fourier Transform, an efficient algorithm for computing the discrete Fourier transform. For all calculations, except for PCA and for obtaining the data displayed in Table 1, images were preprocessed with a lanczos/sinc window function to reduce spectral leakage and wrap around effects.

### Rotational averaging

The 2D power spectra were transformed to 1D power spectra by rotationally averaging the power values for each frequency in the 2D power spectra (Fig. 1), as described previously [12]. Briefly, for each image, data points were binned at regular frequency intervals in the log-log plane. A least-squares fit of a line to these binned data points was performed. Fitting was restricted to the frequency range between 10 and 256 cycles per image, to minimize artifacts due to rectangular sampling, raster screen and low-pass filtering in the image [12]. Results listed in Table 1 are the mean slope of the fitted line and the mean deviation of the data points from that line for each image category (± S.D., respectively).

### Principal component analysis

To analyze the 2D power spectra of the different image categories in more detail, we carried out principal component analysis (PCA) [44], whereby the power spectra of 512×512 pixel size were used as input data. For technical reasons, the 2D power spectrum was cropped squarely so that only the corresponding central part of the power spectrum (256×256 pixels) was used for PCA. Thereby, we excluded high frequencies (<128 cycles/image for vertical and horizontal orientations, to <181 cycles/image for oblique orientations) from the analysis to avoid artifacts (see above), consistent with the rotational averaging procedure. Moreover, frequency components that covered the horizontal and vertical orientations were ignored to exclude continuity and discretization artifacts. The mask used for cropping the power spectrum is illustrated in Figure 2C. The cropped power spectra were used as input data for PCA only. For all other analyses of the 2D power spectra, the frequencies were cropped radially (Fig. 2D). In view of memory and time constraints for computations with high-dimensional vectors, we performed a Lanczos iteration [43] to calculate the strongest eigenvectors and eigenvalues of the covariance matrix, without explicitly computing the covariance matrix itself. In contrast, the low-dimensional vector computations used the sample covariance matrix explicitly.

PCA was performed for images from all categories together, to reveal the components with the largest variance in the input data. The resulting images, displayed in Figure 3, were normalized for visualization, by linearly transforming the minimal and maximal value of the 15 components to the minimal and maximal value of the grayscale range (0–255). To determine the different influences of these components on the diverse categories, a 15-dimensional (15D) eigenspace was computed that was spanned by the coefficients of the 15 leading eigenvectors. In order to quantify how close two image categories are in the 15D eigenspace, the sample covariance matrix was extracted and the mean Mahalanobis distance (Mahalanobis, 1936) was calculated for each pair of image categories. To determine whether categories are distinguishable by the coefficients or not, ANOVA significance testing was performed pairwise for every coefficient on the datasets that contained the coefficients of every category.

To visualize the coefficients of all 15 principal components, the multidimensional input data were displayed as sectors of a circle in a radar plot (Fig. 4). We calculated the means of the positive and negative coefficients separately to capture more of the variance in the data. For each coefficient, data values for all image categories were normalized to the interval [0,1]. The length of the sectors in the radar plot corresponds to the normalized value of the coefficient.

### Determination of anisotropy in the power spectra

To measure anisotropy, the power spectrum was partitioned into 32 radial sectors of equal width (Fig. 2D) [36]. Only the 16 sectors of the top half of the power spectrum were used because the real-valued input images have a symmetric power spectrum. To measure anisotropy of power, we first computed the mean power of every sector with the frequency interval ranging from 10 to 256 cycles per image. Thereby, mean power refers to the mean of a 1D frequency spectrum created by rotational averaging within each sector. To avoid illumination artefacts, we normalized the resulting sector values to a mean of zero and a variance of one. Next, we measured the standard deviation of all sector means for each image (Fig. 5C, H, M, R, W, B', G'; 6A, C). To measure sector anisotropy, we first computed the slope of all rotationally averaged sector spectra. We then measured the standard deviation of all sector slopes for each image (Fig. 5D, I, N, S, X, C', H'; 6B, D). This value is 0 if and only if the slopes are equal in all radial orientations (perfect isotropy), and it increases with increasing anisotropy.

### Psychological evaluation

Six male adult subjects, including two of the authors (M.K. and C.R.), selected 50 images with the strongest perspective lines and depth cues and 50 images with weak or no such cues from the database of monochrome graphic art. The 50 images that were selected most frequently in each of the two tasks were analyzed separately.

Five adult subjects (four males and one female, including two of the authors [M.K. and C.R.]) selected 30 pairs of images from the Groningen database of Dutch natural scenes and from the database of Dutch landscape paintings. The pairs of images from the two databases were matched as closely as possible for similar type of scenery and content. The 30 images from each database that were matched most frequently were analyzed separately.

All subjects had corrected to normal vision. All images from the database were presented simultaneously as prints on paper.

## Supporting Information

Figure S1 Five power spectra from different image categories. A, B/W graphic art; B, B/W portraits; C, face photographs; D, database of Dutch landscape drawings and prints; E, Groningen database of natural scenes; F, Jena database of natural scenes. Examples were randomly chosen from each image database. The low spatial frequencies are represented at the center and darker shades represent higher power.
(7.50 MB TIF)

Figure S2 Five power spectra from different image categories. A, scientific illustrations; B, object photographs; C, plant photographs; D, cartoons; E, comics; F, mangas. Examples were randomly chosen from each image database. The low spatial frequencies are represented at the center and darker shades represent higher power.
(7.23 MB TIF)

Table S1 Pairwise significance testing of the slope value and anisotropy value for all image categories. The p-value of a two-sample t-test is shown for the slope values (upper right half of the table) and the sector anisotropy values (lower left half of the table). Abbreviations: n.s., not significant.
(0.04 MB DOC)

Table S2 Mean Mahalanobis distances between the image categories in a space spanned by the first 15 principal components of 178 spectral features representing each category. Each column represents the distance to one category with the same distance measure.
(0.04 MB DOC)

Table S3 Mean power and sector anisotropies for different subgroups of monochrome art images, calculated separately for different cultural variables. Results for all monochrome art images (art and art portraits) were analyzed together. Values represent mean ± SD (n, number of images analyzed for each category).
(0.05 MB DOC)
