# Supplementary figures and images for: 1/f2 Characteristics and Isotropy in the Fourier Power Spectra of Visual Art, Cartoons, Comics, Mangas, and Different Categories of Photographs
Source: PLoS One. 2010 Aug 19;5(8):e12268. doi: 10.1371/journal.pone.0012268 (PMC2924385; doi:10.1371/journal.pone.0012268)

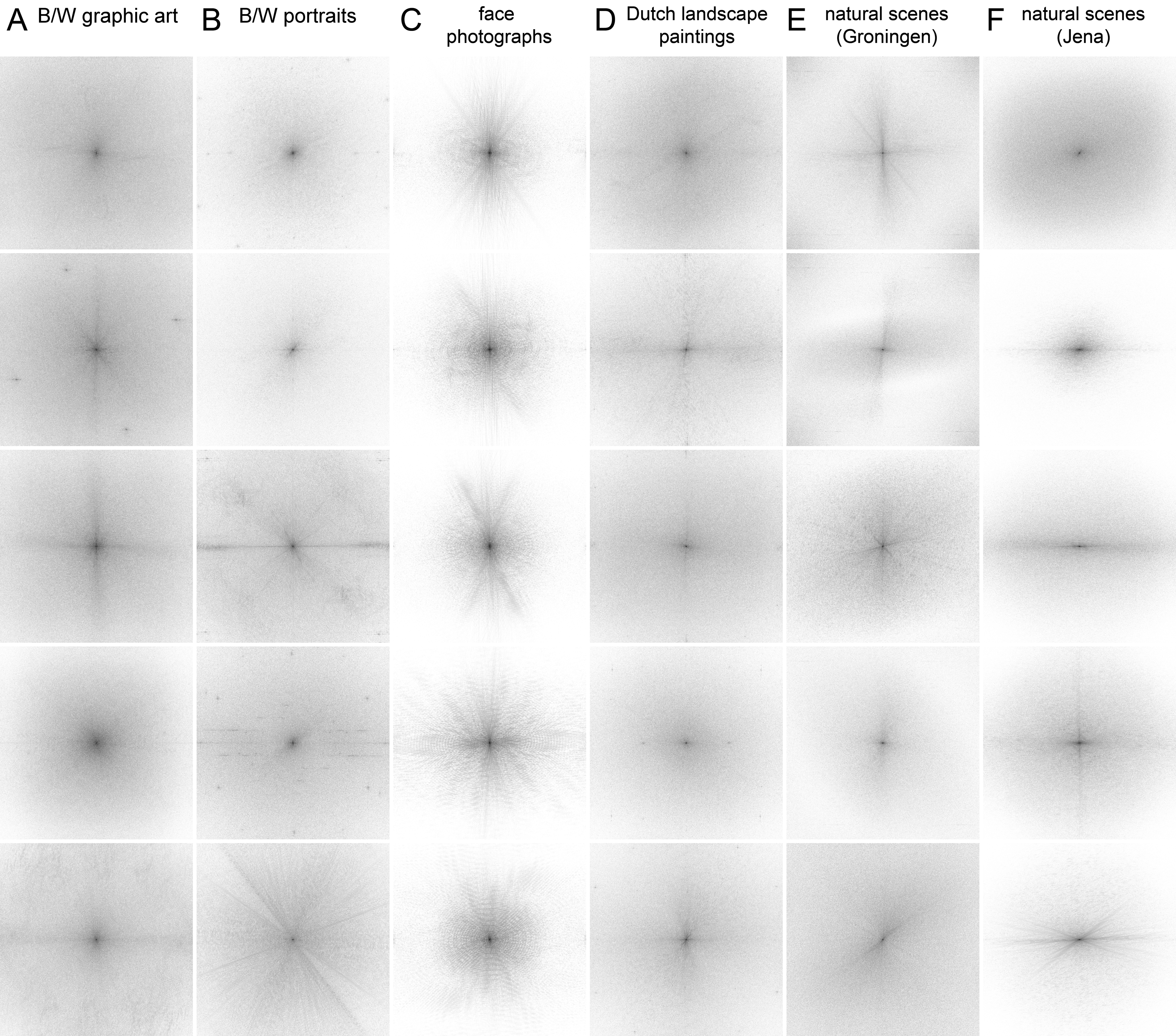

Supplement: Figure S1 — Five power spectra from different image categories. A, B/W graphic art; B, B/W portraits; C, face photographs; D, database of Dutch landscape drawings and prints; E, Groningen database of natural scenes; F, Jena database of natural scenes. Examples were randomly chosen from each image database. The low spatial frequencies are represented at the center and darker shades represent higher power. (7.50 MB TIF) [file pone.0012268.s001.tif]

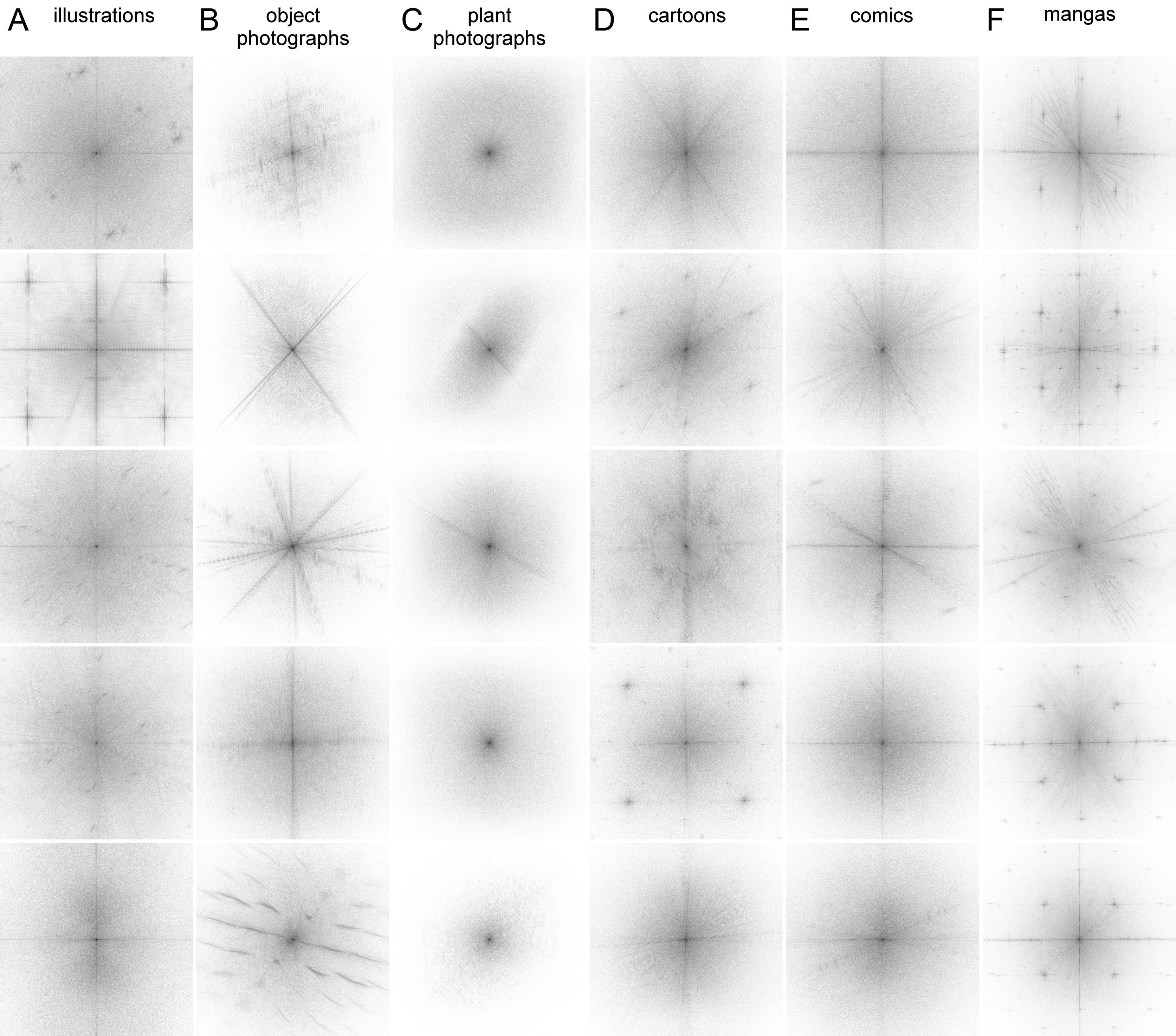

Supplement: Figure S2 — Five power spectra from different image categories. A, scientific illustrations; B, object photographs; C, plant photographs; D, cartoons; E, comics; F, mangas. Examples were randomly chosen from each image database. The low spatial frequencies are represented at the center and darker shades represent higher power. (7.23 MB TIF) [file pone.0012268.s002.tif]
