# Supplementary material for: 1/f2 Characteristics and Isotropy in the Fourier Power Spectra of Visual Art, Cartoons, Comics, Mangas, and Different Categories of Photographs
Source: PLoS One. 2010 Aug 19;5(8):e12268. doi: 10.1371/journal.pone.0012268 (PMC2924385; doi:10.1371/journal.pone.0012268)
Supplement: Table S1 — Pairwise significance testing of the slope value and anisotropy value for all image categories. The p-value of a two-sample t-test is shown for the slope values (upper right half of the table) and the sector anisotropy values (lower left half of the table). Abbreviations: n.s., not significant. (0.04 MB DOC) [file pone.0012268.s003.doc]

|  | B/W graphic art | B/W portraits | face photographs | natural scenes  (Groningen) | illustrations | object photographs | plant photographs | cartoons | comics | mangas | Dutch landscapes | natural scenes  (Jena) |
| --- | --- | --- | --- | --- | --- | --- | --- | --- | --- | --- | --- | --- |
| B/W graphic art | - | < 0.001 | < 0.001 | < 0.05 | < 0.001 | < 0.001 | < 0.001 | < 0.001 | n.s. | n.s. | < 0.01 | n.s. |
| B/W portraits | < 0.05 | - | < 0.001 | < 0.001 | < 0.001 | < 0.001 | < 0.001 | < 0.001 | < 0.001 | < 0.001 | < 0.001 | n.s. |
| face photographs | < 0.001 | < 0.001 | - | < 0.001 | < 0.001 | < 0.001 | < 0.001 | < 0.001 | < 0.001 | < 0.001 | < 0.001 | < 0.001 |
| natural scenes  (Groningen) | < 0.001 | < 0.001 | < 0.001 | - | < 0.001 | < 0.001 | < 0.001 | n.s. | n.s. | n.s. | n.s. | < 0.001 |
| illustrations | < 0.001 | < 0.001 | n.s. | < 0.001 | - | < 0.001 | < 0.001 | < 0.001 | < 0.001 | < 0.001 | < 0.001 | < 0.001 |
| object photographs | < 0.001 | < 0.001 | < 0.001 | n.s. | < 0.001 | - | < 0.001 | < 0.001 | < 0.001 | < 0.001 | < 0.001 | < 0.001 |
| plant photographs | < 0.001 | < 0.05 | n.s. | < 0.001 | n.s. | < 0.001 | - | < 0.001 | < 0.001 | < 0.001 | < 0.001 | < 0.001 |
| cartoons | n.s. | n.s. | < 0.001 | < 0.001 | < 0.002 | < 0.001 | < 0.05 | - | < 0.01 | < 0.001 | n.s. | < 0.001 |
| comics | < 0.01 | n.s. | < 0.001 | < 0.001 | < 0.05 | < 0.001 | n.s. | n.s. | - | n.s. | n.s. | < 0.001 |
| mangas | < 0.002 | n.s. | < 0.001 | < 0.001 | < 0.05 | < 0.001 | n.s. | n.s. | n.s. | - | < 0.05 | < 0.002 |
| Dutch landscapes | n.s. | n.s. | < 0.05 | < 0.001 | < 0.05 | < 0.001 | n.s. | n.s. | n.s. | n.s. | - | < 0.001 |
| natural scenes (Jena) | < 0.001 | < 0.001 | < 0.002 | < 0.001 | < 0.05 | < 0.005 | < 0.05 | < 0.001 | < 0.001 | < 0.001 | < 0.001 | - |
