# Supplementary material for: 1/f2 Characteristics and Isotropy in the Fourier Power Spectra of Visual Art, Cartoons, Comics, Mangas, and Different Categories of Photographs
Source: PLoS One. 2010 Aug 19;5(8):e12268. doi: 10.1371/journal.pone.0012268 (PMC2924385; doi:10.1371/journal.pone.0012268)
Supplement: Table S2 — Mean Mahalanobis distances between the image categories in a space spanned by the first 15 principal components of 178 spectral features representing each category. Each column represents the distance to one category with the same distance measure. (0.04 MB DOC) [file pone.0012268.s004.doc]

|  | B/W graphic art | B/W portraits | face photographs | natural scenes  (Groningen) | illustrations | object photographs | plant photographs | cartoons | | comics | mangas |
| --- | --- | --- | --- | --- | --- | --- | --- | --- | --- | --- | --- |
| B/W graphic art | 0.0 | 3.7 | 23.9 | 1.8 | 2.0 | 2.0 | 4.5 | | 2.4 | 3.2 | 3.5 |
| B/W portraits | 2.4 | 0.0 | 10.8 | 2.4 | 2.6 | 2.1 | 2.1 | | 2.1 | 2.3 | 1.8 |
| face photographs | 3.2 | 3.0 | 0.0 | 3.8 | 3.9 | 2.6 | 2.1 | | 4.1 | 4.4 | 4.4 |
| natural scenes (Groningen) | 1.9 | 4.0 | 22.3 | 0.0 | 2.0 | 2.7 | 4.4 | | 2.5 | 3.8 | 3.4 |
| illustrations | 2.1 | 4.9 | 26.5 | 2.7 | 0.0 | 2.9 | 6.5 | | 2.0 | 3.1 | 4.1 |
| object photographs | 1.7 | 4.9 | 14.1 | 2.9 | 2.3 | 0.0 | 5.8 | | 3.2 | 2.6 | 3.4 |
| plant photographs | 2.1 | 2.0 | 8.2 | 2.4 | 3.4 | 1.7 | 0.0 | | 3.5 | 3.6 | 3.4 |
| cartoons | 1.8 | 2.7 | 14.6 | 2.2 | 1.5 | 2.1 | 4.0 | | 0.0 | 1.1 | 1.8 |
| comics | 1.7 | 3.7 | 12.6 | 2.7 | 1.5 | 1.8 | 4.8 | | 0.9 | 0.0 | 1.6 |
| mangas | 6.7 | 7.8 | 14.6 | 6.7 | 2.9 | 4.6 | 9.2 | | 2.9 | 3.7 | 0.0 |
