# Supplementary material for: 1/f2 Characteristics and Isotropy in the Fourier Power Spectra of Visual Art, Cartoons, Comics, Mangas, and Different Categories of Photographs
Source: PLoS One. 2010 Aug 19;5(8):e12268. doi: 10.1371/journal.pone.0012268 (PMC2924385; doi:10.1371/journal.pone.0012268)
Supplement: Table S3 — Mean power and sector anisotropies for different subgroups of monochrome art images, calculated separately for different cultural variables. Results for all monochrome art images (art and art portraits) were analyzed together. Values represent mean ± SD (n, number of images analyzed for each category). (0.05 MB DOC) [file pone.0012268.s005.doc]

|  | Power anisotropy | Slope anisotropy | n |
| --- | --- | --- | --- |
| All | 0.098 ± 0.039 | 0.18 ± 0.06 | 466 |
| Century | | | |
| 15th Century | 0.097 ± 0.037 | 0.17 ± 0.04 | 33 |
| 16th Century | 0.092 ± 0.036 | 0.17 ± 0.04 | 122 |
| 17th Century | 0.090 ± 0.030 | 0.18 ± 0.05 | 63 |
| 18th Century | 0.098 ± 0.044 | 0.17 ± 0.04 | 33 |
| 19th Century | 0.098 ± 0.039 | 0.18 ± 0.06 | 66 |
| 20th Century | 0.106 ± 0.043^1^ | 0.18 ± 0.07 | 149 |
| Country of origin | | | |
| Italian | 0.095 ± 0.037 | 0.18 ± 0.06 | 99 |
| Flamish | 0.099 ± 0.041 | 0.18 ± 0.06 | 59 |
| French | 0.103 ± 0.039 | 0.19 ± 0.05 | 59 |
| German | 0.096 ± 0.037 | 0.17 ± 0.05 | 197 |
| Spanish | 0.108 ± 0.038 | 0.17 ± 0.08 | 18 |
| Other | 0.108 ± 0.051 | 0.17 ± 0.04 | 34 |
| Technique | | | |
| Etching | 0.101 ± 0.041 | 0.18 ± 0.07^2^ | 114 |
| Engraving | 0.100 ± 0.039 | 0.16 ± 0.05 | 47 |
| Lithograph | 0.104 ± 0.037 | 0.16 ± 0.04 | 42 |
| Woodcut | 0.110 ± 0.044 | 0.17 ± 0.05 | 37 |
| Drawing | 0.094 ± 0.037^3^ | 0.18 ± 0.05^4^ | 226 |
| Subject matter | | | |
| Portrait | 0.090 ± 0.032^5^ | 0.18 ± 0.05 | 317 |
| Landscape | 0.121 ± 0.045 | 0.19 ± 0.07 | 16 |
| Buildings | 0.162 ± 0.037^6^ | 0.18 ± 0.04 | 20 |
| Living matters | 0.106 ± 0.041 | 0.16 ± 0.05^7^ | 78 |
| Abstract | 0.094 ± 0.036 | 0.15 ± 0.06 | 17 |
| Other | 0.125 ± 0.049 | 0.19 ± 0.10 | 18 |

^1^ significantly different from 16^th^ century and 17^th^ century (p<0.01)

^2^ significantly different from lithographs (p<0.05)

^3^ significantly different from woodcuts (p<0.05)

^4^ significantly different from engravings and lithographs (p<0.05 and p<0.01, respectively)

^5^ significantly different from landscapes, buildings, living matters and other subject matters (p<0.05, p<0.0001, p<0.01, p<0.01, respectively)

^6^ significantly different from all other subject matters (p<0.05 to p<0.0001)

^7^ significantly different from portraits and buildings (p<0.001 and p<0.05, respectively)
